# The Role of Transcription Factor 21 in Epicardial Cell Differentiation and the Development of Coronary Heart Disease

**DOI:** 10.3389/fcell.2020.00457

**Published:** 2020-06-05

**Authors:** Haochang Hu, Shaoyi Lin, Shuangshuang Wang, Xiaomin Chen

**Affiliations:** ^1^School of Medicine, Ningbo University, Ningbo, China; ^2^Department of Cardiology, Ningbo City First Hospital, Ningbo, China

**Keywords:** transcription factor 21, development, epicardial cells differentiation, vascular smooth muscle cells, coronary heart disease

## Abstract

Transcription factor 21 (TCF21) is specific for mesoderm and is expressed in the embryos’ mesenchymal derived tissues, such as the epicardium. It plays a vital role in regulating cell differentiation and cell fate specificity through epithelial-mesenchymal transformation during cardiac development. For instance, TCF21 could promote cardiac fibroblast development and inhibit vascular smooth muscle cells (VSMCs) differentiation of epicardial cells. Recent large-scale genome-wide association studies have identified a mass of loci associated with coronary heart disease (CHD). There is mounting evidence that TCF21 polymorphism might confer genetic susceptibility to CHD. However, the molecular mechanisms of TCF21 in heart development and CHD remain fundamentally problematic. In this review, we are committed to providing a detailed introduction of the biological roles of TCF21 in epicardial fate determination and the development of CHD.

## Introduction

The helix-loop-helix (HLH) family of transcription factors possesses highly conserved bipartite domains for DNA binding and protein-protein interactions, which is pivotal in various developmental processes, covering myogenesis, neurogenesis, and hematopoiesis (Jiang and Yang, 2018). The HLH family members are divided into seven families according to tissue distribution, dimerization ability, and DNA binding specificity (Massari and Murre, 2000). Transcription factor 21 (TCF21, 6417bp)—also known as Pod1, bHLHa23, or Capsulin—encodes a transcription factor belonging to the cell-type-specific class II basic helix-loop-helix (bHLH) family at chromosome 6q23.2 (Jiang and Yang, 2018). Moreover, TCF21 is specific for mesoderm and is expressed in the embryos’ mesenchymal derived tissues, such as the epicardium, lung, intestine, gonad, and kidney (Plotkin and Mudunuri, 2008). It regulates the cell differentiation during embryonic development, such as coronary vasculature (Hidai et al., 1998). The ChIP-Seq studies in primary cultured human coronary vascular smooth muscle cells (VSMCs) have identified several TCF21 target genes, such as smooth muscle contraction, growth factor binding and matrix interaction, which are involved in the processes associated with coronary heart disease (CHD) pathophysiology (Sazonova et al., 2015). A wealth of evidence confirms that TCF21 takes an active role in VSMC phenotypic modulation in CHD. In this review, we endeavor to provide a detailed introduction of the biological roles of TCF21 in epicardial fate determination and the development of CHD.

## The Origin of Epicardial Cells

The proepicardial cells, a mesoderm-derived cell cluster, are highly conserved in vertebrates including Xenopus, zebrafish, mice and even human (Cao and Cao, 2018), which could migrate to the myocardial surface, adhere and form the epicardium during embryogenesis (Tandon et al., 2016). Once the original epicardium forms, a portion of the epicardial cells could enter the subepicardial domain and produce epicardium-derived cells (EPDCs) by undergoing epithelial-mesenchymal transition (EMT) (Lie-Venema et al., 2007). During the EMT, epithelial cells downregulate their epithelial gene expression, lose their unique characteristics, and activate the mesenchymal genes to increase cell viability and motility, including aggressiveness (Li et al., 2018). Recent studies have confirmed that EMT could be affected by several signaling pathways in epicardial cells, including but not limited to transforming growth factorβ (TGF-β), retinoic acid (RA), platelet-derived growth factor (PDGF), and extra-cellular matrix (ECM) components (Cao et al., 2020). EPDCs are highly plastic and have been reported to differentiate into myocardial fibroblasts, VSMCs, and pericytes (Gittenberger-de Groot et al., 2010). Lineage tracing studies also confirmed that the VSMCs originated from the epicardial cells (Majesky, 2007).

## TCF21 and Epicardial Cells

### The Role of TCF21 in Epicardial Cells During Heart Development

**FIGURE 1:**
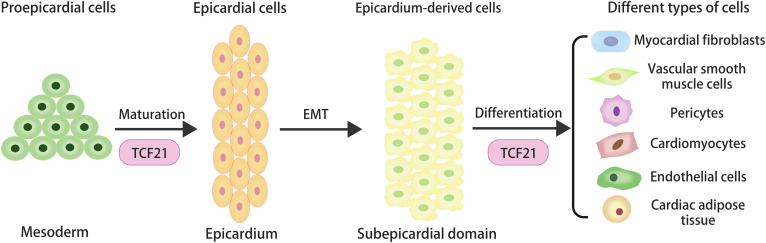
The origin and cellular contributions of epicardial cells during heart development, and the regulatory role of TCF21 in the specification and maturation of proepicardial cells and the determination of epicardial cell fate.

Studies in different animal models have delivered several molecular signatures of proepicardial cells, including the conserved transcription factors [TCF21, Wilms tumor 1(WT1), and T-box factor 18 (Tb×18)] (Cao et al., 2020). The study of Xenopus embryos showed that proepicardial cells could migrate to the heart, retain their precursor cell characteristics and impair maturity in the absence of TCF21 (Tandon et al., 2013). It suggested that the morphological defect in epicardial integrity might be caused by the absence of TCF21 through regulating the specification and maturation of the proepicardial cells at earlier stages of epicardial development (Figure 1).

Previously, we recognized that TCF21 could promote cardiac fibroblast development and inhibit VSMC differentiation of EPDCs. Asha et al. found most epicardial cells expressing TCF21 were lumped in the myocardial fibroblast lineage before the initiation of EMT (Acharya et al., 2012). It was concluded from the study that a TCF21-deficient heart could not undergo EMT and form cardiac fibroblasts, which confirmed the unique role of TCF21 in epicardial EMT and differentiation (Acharya et al., 2012). The *in vivo* experiment showed that loss of TCF21 in mice resulted in a decrease of myocardial fibroblasts due to the premature differentiation of EPDCs into VSMCs through RA signals (Braitsch et al., 2012). Together, these data support the thesis that TCF21 plays an indispensable role in the development of myocardial fibroblasts.

With the development of single-cell sequencing technology, new insights into the differentiation of epicardial cells have been obtained. The near-term single-cell analysis of an epicardium model derived from human pluripotent stem cells showed that the different epicardial subsets were defined by high levels of transcription factors BNC1 or TCF21. In the TCF21 population, TCF21^high^ cells could differentiate into myocardial fibroblasts and VSMCs, while TCF21^low^ cells were mainly limited to VSMCs (Gambardella et al., 2019). These data revealed the complex regulatory effect of TCF21 on fundamental cells fate decisions during heart development. Further studies on the accurate regulatory mechanism of TCF21 in epicardial differentiation should be performed to better explain its role in normal heart development.

### The Role of TCF21 in Myocardial Regeneration

After myocardial infarction, millions of myocardial cells die, which are replaced by fibrotic scar tissue. Regeneration and fibrosis are two important healing processes after heart injury. In response to cardiac damage, epicardial cells can be activated to migrate to the injured myocardium and differentiate into the desired cell lines (especially the cardiac fibroblasts) to support the regeneration of heart tissue (Limana et al., 2007; Bos et al., 2014; Cao et al., 2016). In addition to myocardial fibroblasts, VSMCs, and pericytes, epicardial cells also could give rise to cardiomyocytes, endothelial cells and cardiac adipose tissue during development (Zhou et al., 2008; Zangi et al., 2017). If true, activated epicardial cells can be harnessed to exploit their potential for differentiation to repair injured hearts. However, more evidence needs to be accumulated to prove these assumptions.

Genetic fate-mapping used by Kazu et al. showed that TCF21^+^ epicardial cells of zebrafish might differentiate into perivascular cells, but not to cardiomyocytes during heart regeneration (Kikuchi et al., 2011). In mice with myocardial infarction, TCF21 lineage-traced cells could transform into periostin-expressing myofibroblasts, which is necessary for adaptive healing and fibrosis in the heart (Kanisicak et al., 2016). In addition, the survival rate of myocardial infarction decreases with the reduction of periostin-expressing myofibroblasts, suggesting that fibrosis is an effective mechanism for repairing myocardium. In the past, fibrosis was deemed detrimental to the regeneration process, and it was thought that excessive fibrosis accelerates the progression of diseases. However, fibrosis is transient in zebrafish and fibrosis regression caused by fibroblast inactivation is concomitant with the regrowth of myocardial wall. Sanchez-Iranzo et al., 2018 found that cardiac fibroblasts in zebrafish not only contribute to the fibrosis response after injury but are also necessary for the proliferation of cardiomyocytes during regeneration. All the information on zebrafish’s myocardial regeneration provides a theoretical basis for regenerative medicine. Though regenerative medicine is regarded as a promising treatment for heart disease, many obstacles will have to be overcome before it can be used clinically.

## TCF21 and the Development of Coronary Heart Disease

### Coronary Heart Disease

According to the 2017 National Health Interview Survey, the age-adjusted prevalence of cardiovascular diseases (CVD) was 10.6% (11.0% for Whites, 9.7% for Blacks, 7.4% for Hispanics and 6.1% for Asians) (Virani et al., 2020). Coronary heart disease (CHD), the most common type of heart disease, is featured on the coronary stenosis caused by atherosclerotic plaque (Li et al., 2020), leading to the ischemia and apoptosis of cardiac cells (Firoozi et al., 2020). Acute myocardial infarction (AMI), the most severe consequences of CHD with high mortality, results from prolonged ischemia and irreversible necrosis of the heart muscle (Liu et al., 2009). Therefore, there is a pressing need to elucidate the pathogenesis of CHD and develop an effective therapy for patients with CHD.

As the basic pathological change of CHD, atherosclerosis affects the structure and function of three layers of the coronary artery vessel wall (Wang et al., 2017). Atherosclerotic plaque exists in the intimal space of coronary arteries, between the inner endothelial cell layer and the middle layer of the contractile VSMCs (Truskey, 2010). It is formed by absorbing circulating lipoproteins, recruiting inflammatory cells, and transferring the dedifferentiated VSMCs into the lesion to form a fibrous cap (Nurnberg et al., 2015). In the sections below, we highlight our current understanding of the influence of coronary VSMCs on CHD.

### Phenotypic Modulation of VSMCs

Blood vessels are composed of three layers: the tunica intima, tunica media, and tunica adventitia (Truskey, 2010), with each layer having its unique histological, biological and functional characteristics. The tunica intima, the layer closest to blood, is a continuous lining formed by endothelial cells between the blood and tissues. The tunica media mainly consists of VSMCs and extracellular matrix (circular elastic fibers, collagen I and proteoglycans), which play an important role in maintaining tension and supporting vessels. The tunica adventitia is a loose connective tissue mainly comprising fibroblasts. Myocardial fibroblasts are the main non-myocyte cells in the heart and are critical in maintaining normal cardiac function (Souders et al., 2009).

As an important metabolic and endocrine organ, VSMCs are involved in many physiological and pathological processes through its proliferation, migration, and synthesis of extracellular matrix (Frismantiene et al., 2018). During vascular development, VSMCs secrete major extracellular proteins to keep the unique mechanical properties of vessel walls. Unlike conventional differentiated cells, the phenotypes of VSMCs can undergo reversible changes in different developmental stages, during the progression of diseases or as a response to injuries. The state of VSMCs can be switched between the “differentiated” contractile phenotype and the “dedifferentiated” synthetic phenotype, a process known as phenotypic transformation (Alexander and Owens, 2012). This extensive plasticity of VSMCs gives them the ability to participate in vascular repair.

Indeed, VSMCs principally exhibits the contractile phenotype with low proliferation and synthetic activity, which could regulate blood pressure, blood vessel diameter, and blood flow distribution (Owens et al., 2004), however, the synthetic phenotype loses its contractile function with the low expression of the contractile protein and gains synthetic, migratory, and proliferative properties. Although the phenotypic reversibility is important for normal homeostasis, VSMCs are vulnerable to physiological and non-physiological stimuli that could cause adverse phenotypic switching. A large number of studies have shown that the phenotypic transformation of VSMCs has made a great contribution to various major diseases such as atherosclerosis, asthma, hypertension, and cancer (Alexander and Owens, 2012).

### VSMC Phenotypic Modulation and CHD

Phenotypic modulation of VSMCs is a physiological response to vascular injury. The lineage tracing studies have revealed that about 80 percent of the cells in atherosclerotic plaque lack traditional VSMC marks but about half of these cells originate from the VSMCs (Shankman et al., 2015; Cherepanova et al., 2016). Technical advances in the combination of single-cell RNA-sequencing and lineage tracing have demonstrated multiple dedifferentiated SMC phenotypes in atherosclerosis (Wirka et al., 2019). However, the question of whether VSMC phenotypic transformation in atherosclerosis is protective or deteriorative for the risk of CHD remains unsettled.

On the one hand, under the stimulation of proatherogenic factors, the contractile VSMCs could de-differentiate and be transformed into the macrophage-like inflammatory cells, which could secrete a large number of ECM and inflammatory factors, promoting plaque rupture and acute CHD events (Milutinovic et al., 2020). So, blocking the proinflammatory changes of the VSMC phenotype is of great importance for clinical treatment.

James et al. found that the gene expression associated with the contraction phenotype of VSMCs decreased significantly, while the expression levels of macrophage markers increased after cholesterol loading (Rong et al., 2003). However, MerTK and FcgR1 (CD64), two key marker genes commonly associated with mature macrophages, were not up-regulated in the macrophage-like cells. Cholesterol loading of VSMCs converted them to a macrophage-like state by downregulating the miRNA-143/145-myocardin axis (Vengrenyuk et al., 2015). Notably, these cells were classified as macrophages based largely on single cell-surface protein markers (Pan and Reilly, 2019). However, their transcriptome and functional properties suggested that they act differently from classical macrophages in the formation of atherosclerosis.

On the other hand, VSMCs transition to fibroblast-like cells may enhance the protection of the fibrous cap and reduce CHD events. Notably, the number of VSMCs in the fiber caps was negatively correlated with plaque stability. A thick fiber cap contains plentiful VSMCs surrounding the necrotic core to stabilize the fragile plaque. As the disease progresses, the plaque may rupture violently due to the reduced number of VSMCs in the fibrous cap, leading to arterial thrombosis and acute coronary events (Pan and Reilly, 2019).

In the present study, the single-cell RNA sequencing method was used to characterize the transcriptome phenotype of modulated VSMCs in mice and human atherosclerosis lesions. Though SMC-derived cells could be involved in exocytosis as “non-specialized” phagocytes, it does not have a macrophage-like transcriptional phenotype *in vivo*. Wirka et al. (2019) found that the modulated VSMCs transform into unique fibroblast-like cells, rather than into a classical macrophage phenotype in the ApoE^–/–^ mouse model. Furthermore, the accumulation of VSMCs in the fibrous cap is monoclonal or oligoclonal, which plays a healing role in disease (Orlandi and Bennett, 2010). Therefore, we have reasons to speculate that the VSMCs phenotypic modulation seems to be restorative in atherosclerosis, rather than the primary driver of plaque formation (Bennett et al., 2016).

### TCF21 and VSMC Phenotypic Modulation in CHD

Genome-wide association studies (GWAS) have identified over 160 loci associated with CHD (Wong et al., 2019). There is mounting evidence that TCF21 serves as the causal gene for CHD and that high expression of TCF21 is related to reduced CHD risk in human CHD-related tissues (Wirka et al., 2019). Besides, TCF21 was expressed in human coronary VSMCs but not in aortic SMCs or endothelial cells (Nurnberg et al., 2015). The rs12190287 located within the 3′-untranslated region (3′-UTR) of TCF21 was related to the TCF21 gene expression, which was identified as an expression quantitative trait locus (eQTL) (Schunkert et al., 2011). Race-based research suggested that the SNP of rs12190287 in TCF21 might be an independent genetic risk factor for CHD in Chinese and European patients (Wang et al., 2014; Wirtwein et al., 2017) but not in Japanese patients (Dechamethakun et al., 2014). Another CSMC eQTL variant located in the TCF21 promoter region, rs2327429, was identified as a methylation quantitative trait locus for TCF21 expression, suggesting that methylation regulation is a molecular trait that may mediate the risk of CHD (Gutierrez-Arcelus et al., 2013; Liu et al., 2018). A meta-analysis of two GWAS comprising 1,515 CHD cases and 5,019 controls showed that TCF21 rs12524865 conferred predisposition to CHD in the Chinese and Europeans (Lu et al., 2012). Though significant progress has been made in mapping the genetic contribution to CHD, the molecular mechanisms of TCF21 in CHD remain fundamentally problematic.

**FIGURE 2:**
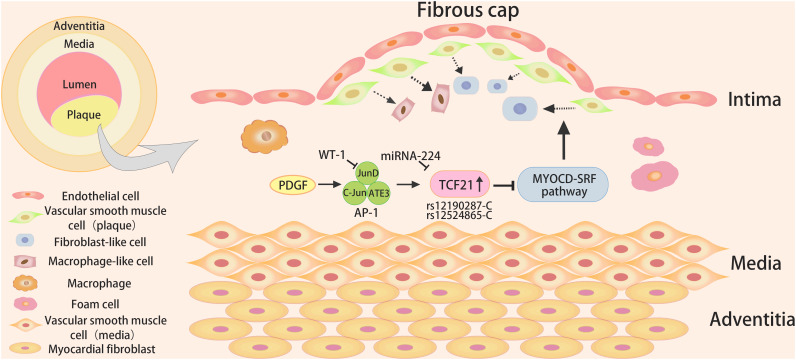
The role and signaling pathways of TCF21 in coronary heart disease. The TCF21 risk alleles (rs12190287-C or rs12524865-C) might result in increased TCF21 expression upon activation of PDGF signaling, and WT1could inhibit AP-1 mediated transcription and c-Jun mediated rs12190287 activation, leading to reduced expression of TCF21. In addition, TCF21 could block the MYOCD-SRF pathway to inhibit the differentiation of VSMCs in atherosclerotic plaque.

It is generally acknowledged that the TCF21 target gene relevant to CHD is enriched in the processes of vascular wall biology (Sazonova et al., 2015). It was observed that VSMC-specific knockout of TCF21 in mice inhibited VSMC phenotypic modulation (Wirka et al., 2019), and the absence of TCF21 results in the decreased fibroblasts in the lesion and the instability of plaque, suggesting that TCF21 functions as a protective factor by promoting VSMC transition to fibroblast-like cells in the lesion and fibrous cap (Figure 2). Regretfully, there are still fundamental questions related to the mechanisms by which it performs this role.

#### The Upstream Pathways of TCF21 in VSMC Phenotypic Modulation

Platelet-derived growth factor (PDGF) signaling mediated by PDGFRβ has been certified as having a major role in EMT, epicardial fate determination, VSMCs migration and proliferation, and atherosclerosis (Smith et al., 2011). The increased PDGF signaling promoted the formation of aggressive plaque in the thoracic aorta and coronary arteries (He et al., 2015). PDGF is a potent inducer of the transformation of contractile VSMCs to secreted phenotype (Raines and Ross, 1993; Owens et al., 2004). Clint et al. found that individuals carrying the TCF21 risk alleles (rs12190287-C or rs12524865-C) appeared to exhibit increased TCF21 expression upon activation of PDGF signaling in coronary VSMCs (Miller et al., 2013). It could increase enrichment of active histone modifications and form an open chromatin conformation, which binds to the atypical AP-1 element, likely including JunD, c-Jun, and ATF3, leading to altering TCF21 expression (Miller et al., 2013). Besides, WT1 as a transcriptional repressor, could inhibit AP-1 mediated transcription and c-Jun mediated TCF21 rs12190287 activation *in vitro* (Miller et al., 2013).

Studies using allelic imbalance sequencing have shown that SNPs could regulate miRNA-mediated repression by altering complementary miRNA binding sites (Kim and Bartel, 2009). The later studies by Clint et al. reported that the major risk C variants of TCF21 rs12190287 preferentially combines with miRNA-224 on account of the altered secondary structure of RNA, ultimately resulting in the downregulation of TCF21 expression (Miller et al., 2014). Furthermore, PDGF signals and TGF-β1 signals are the potential upstream mediators of miRNA-224 to direct the expression of allele-specific TCF21 rs12190287.

Taken together, these two mechanisms happen to explain the two pathways for the alteration of allele-specific gene expression (Mar et al., 2011). A transcriptional regulatory mechanism has been explored that the increased rs12190287 risk C allele could bring about elevated TCF21 expression mediated by PDGF signaling pathways. What is more, the C allele at rs12190287 interacts with miR-224 to reduce the expression of TCF21 through the miRNA mechanism.

#### The Downstream Pathways of TCF21 in VSMC Phenotypic Modulation

Myocardin (MYOCD) is an effective transcriptional co-activator in VSMCs that could bind to the DNA-binding transcription factor serum response factor (SRF) to form a complex and regulate the basic expression program of the VSMC gene (Wang et al., 2001). In addition, MYOCD serves as a major regulator of VSMC differentiation, which could facilitate the expression of VSMC differentiation markers. Manabu et al. used the Assay for Transposase-Accessible Chromatin using sequencing (ATAC-Seq) and ChIP-PCR methods to illuminate that TCF21, co-localized with SRF in the open chromatin region of VSMCs, could inhibit the binding of MYOCD in the human coronary VSMCs at the SRF enhancer (Nagao et al., 2020). Subsequent studies provide evidence for the speculation that TCF21 might disrupt the MYOCD-SRF transcriptional complex and transcriptional regulation of VSMC genes through direct interaction with MYOCD.

Except for the above pathway, Sylvia et al. found that TCF21 could bind to the VSMC marker ACTA2 locus and blocks its expression, indicating a directly inhibiting effect of TCF21 on some VSMC genes (Nurnberg et al., 2015). Also, the downregulated TCF21 expression induced by CXCL12 could decrease the promoter activity of ABCA1 and ABCA1 expression, thus inhibiting ABCA1-dependent cholesterol efflux from macrophages and promoting atherosclerosis (Gao et al., 2019).

It is important to consider that the role of TCF21 in VSMCs phenotypic modulation is regulated by multiple downstream pathways. Additional studies with human vascular disease samples should be conducted to better assess the exact mechanism *in vivo*.

## TCF21 and Other Cardiovascular Disease

Hypertension is the pathogeny of hypertensive heart disease and an important risk factor for CHD. Large-scale association studies showed that TCF21 rs12190287 was a susceptibility locus for hypertension in the Japanese population (Fujimaki et al., 2015). A significant correlation was found between the polymorphism of TCF21 rs76987554 and hypertension (Liang et al., 2017). Previous studies have shown that TCF21 could reduce the cyclin-dependent kinase inhibitor P21 expression in MG63 cells, which is essential for VSMCs proliferation (Funato et al., 2003; Bond et al., 2006), and microvascular stenosis caused by abnormal VSMCs proliferation may play a pivotal part in the occurrence and development of hypertension (Feihl et al., 2008), suggesting that TCF21 might modulate blood pressure through p21-dependent microvascular remodeling. Caitlin et al. found that in the advanced stages of the disease, the predominant expression of TCF21 occurred in perivascular fibrosis caused by pressure overload in the adult mice (Braitsch et al., 2013). Therefore, we inferred that TCF21 might contribute to the genetic susceptibility to CHD by affecting the predisposition to hypertension.

Congenital heart disease is an occasional, isolated, non-syndromic defect that may be the result of an interaction between genetic and environmental factors (van der Linde et al., 2011). Ventricular septal defect (VSD) is one of the most common types of congenital heart disease. Recent research has shown that allele G at variant rs12190287 is a contributing risk factor for VSD in the Chinese population (Yang et al., 2017). Together, all these data indicated that TCF21 might confer genetic susceptibility to cardiovascular disease.

## Conclusion and Prospects

In conclusion, the abovementioned studies demonstrated that TCF21 was engaged in the specification and maturation of proepicardial cells and the fate determination of epicardial cells during cardiomyogenesis. And TCF21 could activate the protective role of VSMC phenotypic modulation via the complex upstream and downstream signaling pathways in CHD. Both PDGF signaling pathway and miRNA mechanism might be potential mechanisms for the regulation of TCF21 expression. Moreover, TCF21 could modulate VSMCs response to vascular stress and injury by antagonizing the MYOCD-SRF pathway. Further investigation should be undertaken to explore whether and how TCF21 interacts with other factors to influence VSMC phenotypic modulation.

Recent evidence has confirmed the momentous role of TCF21 in epicardial cell differentiation, and it has been found that TCF21 rs12190287 polymorphism is associated with VSD, a congenital heart disease. Therefore, we speculated that TCF21 might play a regulatory role in heart development and related diseases. The study of the corresponding mechanism principle will be of great interest in the future.

The single-cell sequencing studies have shown that VSMCs transitioned almost exclusively to fibroblast-like cells, but not to macrophage-like cells in animal models with 16 weeks of disease progression (Wirka et al., 2019). Furthermore, the pro-differentiation related factors, such as SMAD3 and TGFβ1 could increase the risk of CHD (Miller et al., 2016; Iyer et al., 2018), which reinforces the evidence in reverse. If this is the case, it might clarify the mainly anti-atherosclerotic effect of VSMCs, thus providing strong evidence for treatment. Based on the flaws in current research, such as short observation time and defect in the detection method, a combination of complementary methods should be used to verify the atheroprotective role.

## Author Contributions

XC, SL, and HH contributed to the conception and design of manuscript. HH, XC, and SW contributed to the writing and final approval of the submitted revision.

## Conflict of Interest

The authors declare that the research was conducted in the absence of any commercial or financial relationships that could be construed as a potential conflict of interest.
